# What is Causal Specificity About, and What is it Good for in Philosophy of Biology?

**DOI:** 10.1007/s10441-021-09419-x

**Published:** 2021-07-12

**Authors:** María Ferreira Ruiz

**Affiliations:** Department of Philosophy, Universitat Bielefeld, Bielefeld, Nordrhein-Westfalen, Germany

**Keywords:** Causal parity thesis, Causal specificity, Causal selection, Genetic causation, Fine-grained control

## Abstract

The concept of causal specificity is drawing considerable attention from philosophers of biology. It became the rationale for rejecting (and occasionally, accepting) a thesis of causal parity of developmental factors. This literature assumes that attributing specificity to causal relations is at least in principle a straightforward (if not systematic) task. However, the parity debate in philosophy of biology seems to be stuck at a point where it is not the biological details that will help move forward. In this paper, I take a step back to reexamine the very idea of causal specificity and its intended role in the parity dispute in philosophy of biology. I contend that the idea of causal specificity across variations as currently discussed in the literature is irreducibly twofold in nature: it is about two independent components that are not mutually entailed. I show this to be the source of prior complications with the notion of specificity itself that ultimately affect the purposes for which it is often invoked, notably to settle the parity dispute.

## Introduction

Traditionally, the philosophy of causation has put the focus on the project of distinguishing causes from non-causes by clarifying what it is to be a cause. Recently, the complementary project of distinguishing among causes is receiving considerable attention as well (Woodward 2010). This latter task makes use of additional causal concepts that characterize differences in causal contributions, enabling comparisons among them. This is an important philosophical project for clear reasons. Often, one is not exclusively concerned with *detecting* causal relations, but with the characteristic features of particular causal contribution. Moreover, we often acknowledge a given effect to have multiple causes, so the question arises as to how they compare in terms of diverse characteristics. A theory of causality that merely tells us how to distinguish causes from non-causes would not help us answer that question. Analyses of the respects in which causal relations differ can be done on the basis of several properties (e.g. strength, proportionality, stability), but I will be concerned with one that has inspired interesting disputes in the philosophy of biology, namely *causal specificity*.

The notion of causal specificity has been spelled out in a number of ways; for Waters (2007), for instance, specificity is about the possibility that many different changes in the cause would produce many different changes in the effect. Philosophers of biology have invoked causal specificity to make the case that genetic and nongenetic developmental factors are not causally on a par, in other words, to argue against the causal parity thesis. The general rationale of specificity-based arguments is that it does not follow from the fact that there are multiple causes for a given effect that they are the same, ontologically speaking (i.e. qua causes). Thus, opponents of causal parity have invoked the concept of causal specificity to make the case that DNA is ontologically different. However, it has been replied that non-genetic factors can be highly specific as well.

Regardless of the stance taken with respect to the causal parity of developmental factors, there is a consensus view that specificity captures an interesting and distinct feature of causal relations. In this context, two things seem clear to me. First, the debate around causal parity in philosophy of biology is a bit stuck and, at this point, it is not more biological details that will help fix the situation (as, in fact, opposing conclusions have been drawn from biological details). Second, philosophers seem to assume that attributing specificity to causal relations is at least in principle a straightforward (if not systematic) task, and that causal specificity is a perfectly coherent, intuitive, well understood notion.

In this paper, I take a step back to reassess the value and limitations of the notion of specificity for the dispute. This is not carried out on the basis of further analysis of the overly discussed cases of DNA vs. the polymerase, or vs. alternative splicing. Rather, I point to *prior* difficulties with the notion of specificity itself and show that they ultimately affect the purposes for which specificity is often invoked, notably to settle the parity dispute. I argue that causal specificity, in all available accounts, is really about two distinct components, *connectedness* and *repertoire*, which—crucially—are not mutually entailed. Furthermore, I identify this dual nature as the source of the difficulties for attributions of causal specificity to any given causal relation. While my analysis has direct consequences for a particular debate in philosophy of biology that are made explicit in the paper, it will be of a broader interest in the philosophy of causation.

The paper is structured as follows. In Sect. 2, I present the causal parity thesis in philosophy of biology, clarifying the principles upon which it rests. I then outline the basic logic of the specificity arguments against causal parity, illustrating how specificity works there. In Sect. 3, I review the ways in which causal specificity has been spelled out in the literature. Next, I address the question as to whether specificity should be viewed as a quantitative or as a categorical property, and recommend the former option following some of the suggestions in the literature (Sect. 4). In Sect. 5, I raise a paradoxical reading of the so-called “switch-like” causal structures in order to reveal the dual nature of causal specificity and clarify its components and their mutual independence. This is identified as the source of the difficulties explained in Sect. 6 concerning the specific/nonspecific distinction and how to attribute causal specificity. The consequences of such difficulties for the parity dispute in philosophy of biology (the success of specificity-based arguments), and ways to deal with those difficulties, are examined in Sect. 7. Lastly, some conclusions in Sect. 8.

## Causal Parity and the Specificity Argument

Parity claims in biology are mainly attributed to Developmental Systems Theory, a challenge to traditional dichotomous, gene-centric views of development and evolution (for good overview, see Oyama et al. 2001). According to this tradition, biological phenomena can be accommodated along a series of dichotomous categories: nature/nurture, inherited/acquired, genetic/environmental; where the latter is spelled in terms of a few others: information carriers/material support, replicators/interactors, controllers/controlled, instructive/permissive, etc. Developmental Systems Theory is an attempt to move beyond the traditional dichotomous view on the grounds that it fails to account for the complexity of biological phenomena.

The talk of *parity claims* as such is not so widespread. The literature typically mentions “causal parity” or “the causal parity thesis”, and often fails to distinguish among sorts of parity (an exception is Stegmann 2012). At least three senses of parity are distinct enough to call fairly independent analyses: causal, informational, and methodological (Ferreira Ruiz 2019). Here, we are only concerned with causal parity.

Advocates of causal parity (sometimes also called “symmetry”) reject any principled, metaphysical, objective distinction between genetic and nongenetic causes^1^ (e.g. Oyama 1985; Griffiths and Gray 1994; Griffiths and Knight 1998; Griffiths and Stotz 2013). Causal parity claims are motivated by the causal complexity of biological phenomena and emphasize that such phenomena are typically brought about a multiplicity of (interacting) causes. Taking RNA synthesis as an example, causal parity (minimally) highlights the fact that DNA is not causally sufficient to bring about the synthesis of RNA molecules; rather, a particular cellular milieu and a variety of proteins and other molecules are needed for synthesis to take place. Typically, parity advocates commit to the following tenets:

**T1:** *Multiplicity of causal factors:* Biological phenomena are attributable to an assembly of multiple causal factors.

**T2:** *Insufficiency of single causes*: No developmental factor alone is sufficient for the effect.

**T3:** *Metaphysical homogeneity of causal roles*: However causal roles may differ, they do not differ metaphysically.

T1 and T2 are thoroughly addressed in (Oyama 1985). T3 is especially articulated in an attempt to counter objections that the idea of parity obscures valuable distinctions:

The 'strawman' parody of developmentalism says that all developmental causes are of equal importance. The real developmentalist position is that the empirical differences between the role of DNA and that of cytoplasmic gradients or host-imprinting events do not justify the metaphysical distinctions currently built upon them (Griffiths and Knight 1998, p. 254; see also Griffiths and Gray 1994, p. 277).

At first glance, it is unclear what such “metaphysical distinctions” would amount to^2^, but we get a better grasp of the causal parity thesis in the light of its criticisms, which invoke causal specificity.

Thus, let us now consider the rationale of anti-parity arguments. (For the ease of exposition, the meaning of specificity here will be bracketed until the next section). Causal specificity appears in the causal parity dispute in biology as a result of the way this thesis has been interpreted by its main critics. While virtually everyone would accept T1-T2 above (as do critics of causal parity), T3, on the other hand, is contentious.

Most replies to parity interpret T3 in connection with the problem of causal selection. This longstanding problem of concerns the grounds for singling out one or a few factors as ‘the cause(s)’ of an effect while relegating others to ‘background conditions’ (Broadbent 2008; Franklin Hall 2015; Ross 2018). Provided that causal explanation is typically selective in this sense, both in everyday causal judgement and in scientific practice (as we never cite *every* factor relevant to an effect), the question arises as to whether this practice of selecting causes responds to objective or only to pragmatic, interest-laden criteria. Here, some take it that the standard view in philosophy is that which can be traced back to John Stuart Mill (Mill 1974 [1843], book III, ch. V). According to Mill, the way we select causes from among various conditions is interest-laden (p. 329).

Thus, T3 would express a Millean view that the only objective distinction is between causes and noncauses. Critics of causal parity reject such Millean view, and contend that it does not follow from the fact that there be multiple causes that they be ontologically the same (“the fallacy of parity arguments” in Waters 2007).

Opponents of the causal parity thesis have invoked the concept causal specificity to make the case that DNA is *ontologically different*. This means that the customary singling out of genetic factors does not merely follow from the needs and interests of particular investigations, but it (in addition) reflects some objective aspect of the world. Once an effect has been specified, the question about its causes is an ontological one. Similarly, once the causes of a given effect have been identified, the issue of their characteristics (here, whether they bear a specificity relation to the effect) is, too, an ontological one.

Specificity-based arguments against the causal parity thesis posit an (objective) difference that is either *categorical*, that is, whereby some factors (DNA, and perhaps some other) *are* causally specific with respect to (for example) RNA synthesis but others are *not*, in a clear-cut way; or settle for a *quantitative* type, such that DNA is causally *more specific than* other types of factors. Having presented the wider context of the discussions, we can now turn to the available formulations of specificity.

## Specificity, a Few Ways

First, some working distinctions are in order. Indeed, because specificity is a key and pervasive concept in biology, we need to avoid easy misunderstandings. Very roughly, we can identify a *biological sense* and a *philosophical sense* of specificity. In the first case, we are talking about specificity as used by biologists to refer to distinct *biological phenomena*^3^. Even when the biological uses of specificity can, as it happens, be further analyzed philosophically, we can conceive of them as distinct –at least, in the sense an explicandum and its explicatum are distinct (Carnap 1950)^4^.

In the second case, we are talking about *causal concepts*, that is, concepts for features of causal relations that are the object of philosophical enquiry. This is the sense of specificity features in the causal parity debate, and has to do, in a nutshell, with the possibility that many different changes in the cause led to many different changes in the effect. When causal relations have this property, they can be exploited in various ways, allowing a fine-grained control of what happens with the effect (this is why it is also referred to as “fine-grained influence”).^5^

In addressing the problem of causal selection, Kenneth Waters (2007) proposes that practices of causal selection in biology are often guided by the identification of the cause that *actually made a difference*, relative to a given population. However, he acknowledges that this alone fails to account for biologists’ singling out of DNA in the context of protein synthesis, because factors other than DNA that are not similarly selected constitute actual difference makers *too*. The case requires, then, noting that not all actual difference makers are equal: some are causally specific. By this, he means that different changes in the sequence of nucleotides in DNA would change the linear sequence in RNA molecules in many different and very specific ways. Generalizing,

**Specificity (Waters) **Many different changes in the cause produce many different changes in the effect.

This is contrasted with the role of the RNA polymerase, as another salient factor that participates in the same process and contributes to the same effect (RNA synthesis) as DNA. Waters contends that the polymerase lacks this specificity because it is not the case that many different changes in this enzyme will lead to many different changes in the RNA product. Rather, such changes would slow down or stop the RNA synthesis altogether.

 Waters acknowledges that the idea resembles David Lewis’ notion of influence. In Lewis’ (2000) view, roughly, influence is a matter of there being “not too distant” alterations of the cause and the effect, which are connected counterfactually:

**Specificity (Lewis) **C influences E iff there is a substantial range C1, C2,… of not too distant alterations of C, and a range E1, E2… of alterations of E, at least some of which differ, such that if C1 had occurred, E1 would have occurred, and if C2 had occurred, E2 would have occurred, and so on.

A fundamental difference between the two concepts here is that Waters takes specificity to characterize certain causal relations, while Lewis’ influence was aimed at characterizing causal relations *simpliciter* (and fails to do so according to a broad consensus in the philosophy of causality literature, see e.g. Schaffer 2001).

A different definition is provided by James Woodward (2010) within the interventionist framework. He claims to clarify Waters’ notion of specificity by drawing on Lewis’ notion of influence. The combination of both within the interventionist framework and terminology results in an idea of specificity as fine-grained control, which is presented intuitively with a radio analogy that compares its *on/off switch* to its *tuning dial*. There are many possible positions for the dial, many possible radio stations, and a relation holding between both that enables a fine-grained control over what is heard on the radio. I can intervene on the dial in many ways so as to tune various different stations. By contrast, the on/off switch, while causally relevant to whether *a* station is received, it has little influence on *which* one is received. One cannot intervene on the on/off button in order to tune various different stations, but only to turn the radio either on or off. Woodward defines specificity as follows (slightly simplified):

**Specificity (Woodward) **There are a number of different possible states of *C* (*c*1… *c*n), a number of different possible states of *E* (*e*1… *e*m) and a mapping *F* from *C* to *E* such that for many states of *C* each such state has a unique image under *F* in *E* (that is, *F* is a function or close to it, so that the same state of *C* is not associated with different states of *E*, either on the same or different occasions), not too many different states of *C* are mapped onto the same state of *E* and most states of *E* are the image under *F* of some state of *C*. *F* should describe patterns of interventionist counterfactual dependency.

This definition is meant to allow for a better characterization of biological causes and, thus, a better comparison among them (Weber 2017a contains the most elaborated argument based on the notion above). Claims that DNA is a highly specific cause (or a dial-like cause) of protein synthesis mean that: “there are many possible states of the DNA sequence and many (although not all) variations in this sequence are systematically associated with different possible corresponding states of the linear sequences of the mRNA molecules. (…) Thus, varying the DNA sequence provides for a kind of fine-grained and specific control over which RNA molecules or proteins are synthesized” (p. 306). Claims that the polymerase is not specific mean that interventions on it do not provide fine-grained control. The role of the RNA polymerase, by contrast, switch-like.

In the case of maximal specificity (‘ideal’), the mapping is a 1-1 function (every value of the effect variable maps onto one value of the cause variable, and vice-versa). Woodward notes that this might not be the rule in real-life cases, as they are typically not bijective. In such cases, he contends, we have *more* specificity the closer the mapping gets to a bijection (i.e., a one-to-one mapping). This is an explicit proposal that specificity is a property that comes in degrees (but Weber 2006 first suggested that this could be the next step). Waters’ treatment of DNA and nongenetic causes seems to attribute either specificity or lack of specificity, and it can be odd to regard Lewis’ influence as admitting of degrees, if the notion characterizes causation simpliciter (perhaps leading to the contentious idea of graded causation). In any case, Woodward’s quantitative notion was taken seriously enough to motivate a measure of causal specificity.

In fact, Paul Griffiths et al. (2015) put forward a measure of causal specificity (in the sense of fine-grained control above) based on the formalism of Shannon’s mathematical theory of information (1948). Their motivation is that the previous literature on causal specificity is mostly “qualitative”, and that the notion had not been made “adequately precise”. Thus, they claim, “a merely intuitive approach to causal specificity is unlikely to be helpful in settling disputes like this (p. 531). The idea is that we can measure how much knowing the value set by an intervention on a causal variable reduces our *uncertainty* about the value of an effect variable. To know this, one needs to compare the entropy (or information) of the probability distribution of the values of the effect variable *before* and *after* intervention on the cause. The greater the difference in these entropies, the more uncertainty is reduced by intervening on the cause, and the more specific the relation is.

**Specificity (measure) **The specificity of a causal variable is obtained by measuring how much mutual information interventions on that causal variable carry about the effect variable.^6^

Three quantities are key in this framework: the entropy of the probability distribution of the effect values, $\rm {H}\left(\rm {E}\right)$; the conditional entropy of $E$ having set the value of the cause by intervention, denoted with a hat, $\rm {H}(\rm {E}|\widehat{\rm {C}})$; and the mutual information between cause and effect having intervened on the cause, which obtains as $\rm {H}\left(\rm {E}\right)-\rm {H}(\rm {E}|\widehat{\rm {C}})$. In this way, causal specificity becomes identified with mutual information between interventions on the cause and effect: it tells us how much an intervention on a cause specifies an effect^7^. This measure is used to show that alternative splicing factors bear a degree of specificity comparable to that of DNA with respect to RNA sequence. Notably, the same measure has been used to show (without denying the specificity of alternative splicing factors) that DNA still scores higher in specificity in this sense (Weber 2017b).

While the characterization of causal specificity has been refined and improved, we will see that crucial issues that remain and have been overlooked. Having depicted the rationale of the specificity argument and reviewed formulations of specificity, a question arises as to what sort of distinctions do the parity and anti-parity stances need or posit. This will prove relevant to the remainder of the paper, for reasons that will become clearer in the next section.

## What Kind of Distinctions does Specificity Enable?

When we consider the question as to the sort of distinctions that are at play here (and as mentioned in passing in Sect. 3), two options come to mind: either specificity licenses categorical distinctions, or it enables distinctions in degrees. In the latter case, I will refer to specificity as a quantitative property, covering both views that yield comparative ascriptions (‘A is more/less specific than B’) and views that allow assigning a numerical value (‘A scores 1 bit in specificity’, e.g. following the measure above). This choice is generally relevant in the parity debate, as it bears on the strength of anti-parity claims. More importantly, it will prove relevant to this paper, as I will make the case that the difficulties with causal specificity that affect its use in parity arguments arise *even* for what seems to be the most plausible option, namely the quantitative distinctions.

Thus, is the specific/nonspecific distinction meant to be quantitative or categorical? What sort of specificity-based distinction is rejected by the causal parity thesis and defended by opponents? One option is to consider the relevant distinction to be categorical, this is, where causal parity requires showing that there are no categorical differences between biological factors, and non-parity requires showing that categorical distinctions are possible. Griffiths and Gray’s claim above about there being “nothing that divides the resources into two fundamental kinds” seems to go along this line. There is no property of developmental resources on the basis of which we could draw a sharp distinction. The way Waters treat the DNA vs. polymerase situation seems to accept exactly these terms for the discussion: DNA *is* specific, the polymerase *is not*. This sounds like a categorical distinction. The radio analogy, in turn, reinforces this contrast: dials and switches are different *kinds* of things.

However, more recent contributions to the debate (from both parties) seem to have shifted towards a quantitative point of view. Note that quantitative here should cover both the measure and a comparative concept (“more/less specific than…”). Woodward’s notion of specificity (and hence specificity-based distinctions) is explicitly quantitative at least in a comparative sense: in real-life cases where the mapping will typically not be bijective, we have more specificity the closer the function gets to a bijection. (I have doubts that the radio analogy, introduced by Woodward himself, is the best way to capture this notion of degrees of specificity. I turn to these issues in Sect. 7). The information-theoretic measure of causal specificity is another straightforward case where specificity is conceived as coming in degrees. In this case, specificity is not merely a comparative concept: we obtain numerical values for the degree of specificity.

Now, what does the causal parity debate look like, from such quantitative angle? The point of causal parity would be to emphasize that different factors exhibit *the same* causal property, and put less weight on the degree to which this is so. They would hold that specificity cannot elevate a factor to a special status, or single it out, either because differences in specificity are negligible (which is an empirical matter and depends on the case) or because differences in degree would not in general ground the sort of ontological claim that non-parity is supposed to be. Opponents of the causal parity thesis, on the other hand, would put the emphasis elsewhere. They would stress how various causal factors exhibit a property *to varying degrees* despite it being the same. Consequently, they would hold that specificity does single out certain factors, that differential degree is what matters, and that this differential degree allows for exactly the sort of ontological differences that the stance requires.

The recent discussion between Weber (2017a, b) and Griffiths et al. (2015) shows exactly this dialectic. The latter propose and apply the measure hoping to show that alternative splicing factors can be nearly as specific as DNA with respect to RNA synthesis. In turn, Weber (2017b) uses the same measure to show exactly the opposite: that even if other, non-genetic factors bear a specific relation to some effect, it will never exceed the degree of specificity characteristic of DNA. The discrepancies here stem from various decisions concerning the application of the measure to a concrete, particular case. Notably, it depends on the *range of variation* that should be considered, and on how the DNA causal variable is construed in the comparisons^8^. As much as these decisions are relevant, they should not give the impression that this is the extent of the problem. On the contrary, problems arise even under full agreement as concerns such decisions.

Indeed, I will explain that the specific/nonspecific distinction *made quantitative*, while rings more plausible than the categorical alternative, is nevertheless itself puzzling (this is, not as a consequence of particular applications to concrete cases). This can now turn to the twofold nature of causal specificity.

## Two Components of Causal Specificity

As things stand now, there is some tacit consensus that we have a good grasp of the specific/nonspecific *distinction*, at least at the level of intuitions. There is also consensus that this notion captures an interesting and distinct causal feature, notably but not exclusively in biology. In fact, recent disagreements featured in the literature stem more from diverging assessments of concrete cases, and/or different ways to implement the concept/measure in those cases, than from ambiguities or issues *with the notion itself*. I will now articulate some issues of the latter kind, centered around the specific/nonspecific distinction.

The quantitative shift in the specificity debate, as reviewed above, favors the picture of a specificity gradient, ranging from minimum to maximum specificity. This idea seems prima facie unproblematic, as quantitative properties are ubiquitous. It might even seem more plausible than a qualitative/categorical view of specificity. So, the literature suggests the following picture, where switches are to be placed in the minimum extreme of the gradient:

However, on a closer inspection, it is far from obvious that the switch-like cases (recall: 2 values for each variable, plus a bijective function relating them) should be placed there, in the minimum extreme of the specificity gradient rather than in the opposite one. The reason why this happens is the very nature of specificity, as we will now see.

I submit that specificity, in every account, is a dual causal notion, this is, one about two components, that I will call *repertoire* and *connectedness*. Assuming the relevant relations are causal, and expressed here as terminologically neutral as possible, these components impose the conditions that:

**REPERTOIRE** *many* possibilities exist on each side (cause; effect), and

**CONNECTEDNESS** that the possibilities on both sides are connected *in a particular, relevant way*

The formulations here are intendedly vague (“many”, “in a particular or relevant way”) for the sake of terminological neutrality, but we might as well turn to the Woodwardian terminology to express these ideas. REPERTOIRE refers to the number of different values that an effect variable and a cause variable can take. Note that CONNECTEDNESS is not simply about imposing a connection simpliciter (this would be trivial, as we are already dealing with causal relations). And it is also not about particular activities, actions, or the “special causal concepts” in an Anscombian sense (e.g. ‘scratch’, ‘push’, ‘burn’ (Anscombe 1971)). Rather, CONNECTEDNESS is about a further condition on the causal connection, irrespectively of the type of action. In Woodwardian terms, it can be expressed as a condition for the *function* connecting values of the cause and effect variables (in particular, imposing that the function be bijective, or similar).

We can bring the radio example for a simple illustration. Suppose the relevant effect (what I wish to have a fine-grained control over) is the music for my car ride. (We must assume, for the sake of the example, that the following is a good causal description of the situation, e.g., that the level or grain of description is accepted as appropriate). Three scenarios can be compared.

In a first scenario, my tuning dial that can take up about 20 positions and I have about 20 music options to choose from within my reach. This is a typical situation where each position in the dial corresponds to one radio station, so we have here a one-to-one mapping between values of the cause (dial positions) and values of the effect (music options). Now compare that situation to one where my dial has only four possible positions, and I am driving around a small village where I can only pick up four stations broadcasting music. The two scenarios show the same mapping of cause and effect; the difference here lies in the REPERTOIRE of both causal relata. Consider now a third situation we where we have again a 20-position dial. Suppose that five of these allow me to tune five different radio stations, transmitting various different kinds of music, but there is one program, “The best of Genesis” that is (for some reason) broadcast on 12 different stations. A country music program is, similarly, broadcast on three. There will be 12 different positions of the dial that would lead me to listen to exactly the same Genesis songs, three positions that will give me the same country music songs, and five remaining positions with which I can tune five different programs. In both the first and third scenarios, we have 20 possibilities for the dial. The difference between these two, then, corresponds to a difference in CONNECTEDNESS.

To be fair, there has been some recent recognition that causal relations might differ, in terms of their degree of specificity, in respects involving what I call REPERTOIRE and CONNECTEDNESS (see Stegmann 2014 and Weber 2017a)^9^. However, two things are missing in previous work on specificity. First, the distinction has not been directly addressed and, as a consequence, the question has not been posed whether such duality is essential to the notion, or simply a drawback from particular definitions that happen to be inadequate, but which could be amended. Secondly, previous recognition that specific relation might differ in two respects has not been followed by reflections on the problems this leads to. However evident the dual nature of specificity is (if evident at all), it has *not* been identified as problematic for the coherence or, at least, the operationalization of our causal property.

Indeed, an important fact concerning these two components has been overlooked, namely that they are quite independent. Yet it is not difficult to observe that they are not mutually entailed:

- REPERTOIRE does not entail CONNECTEDNESS: the existence of many possibilities does not necessitate a particular type of connection (e.g. a bijective function),
- CONNECTEDNESS does not entail REPERTOIRE: the existence of a particular type of connection does not necessitate any particular number of possibilities^10^.

The only link between the two components is that this is how, *as a matter of fact*, we seem to reason about causal specificity and, to this extent, is constitutive of the idea of causal specificity. It seems that by focusing on only one component at a time, causal specificity would remain undefined.

Having identified the two (independent) components of specificity, the point I will make next is that this dual nature of causal specificity threatens the coherent attribution of specificity and raises issues that affect the purposes for which it is often invoked, as I will explain.

## Attributing Causal Specificity

**Fig. 1:**
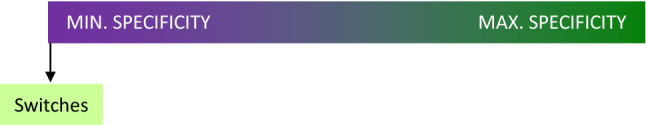
A specificity gradient showing the customary conception of switch-like causes

As we saw in Sect. 4, we can conceive of specificity arguments where specificity is either a categorical property or one that comes in degrees (either measurable or comparative). Spelling out the specific/nonspecific distinction as categorical would prove extremely difficult, and perhaps unnecessary, as we have clearer formulations of specificity that render it quantitative. Here, I will show that the attribution of causal specificity, and comparing degrees of specificity under a quantitative angle is hampered by the independence of the two components of specificity (Fig. 1).

**Fig. 2:**
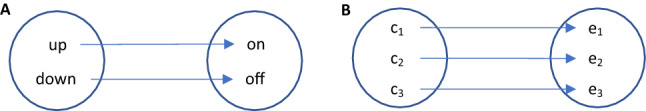
A switch-like structure (**a**) and another bijective structure featuring an additional value for each variable

**Fig. 3:**
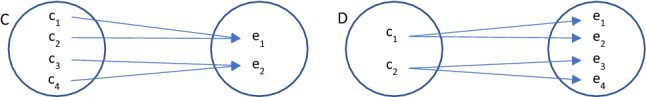
Two different non-bijective cases

We can start by taking a closer look at the switch-like cases. **A** in Fig. 2 below corresponds to what is called a switch: two values for the causal variable (up; down), two values for the effect variable (off; on), and a bijective mapping from cause to effect. Because the two components of specificity are not mutually entailed, depending on which component is given more weight, intuitions regarding the specificity we should ascribe to switch-like cases will vary (Fig. 3).

Indeed, switch-like cases can be considered either *minimally* specific, because there are very few possibilities, or *maximally* specific, because the function is bijective. This seems paradoxical, and perhaps even points to causal specificity being ill-defined. If a switch is an extreme case of a dial (rather than something categorically distinct), in *which* sense is a switch an extreme case? Arguably, these cases rank low in REPERTOIRE and high in CONNECTEDNESS. In the specificity debate, however, **A**-cases are introduced as contrasting with highly specific causal relation, this is, introduced as the opposite of a dial. But this must be acknowledged to work under the assumption that it matters more, if not exclusively (for the conclusion), that it scores low in REPERTOIRE. And the difficulty arises if we look at other types of situations, too. Consider case **B**: what should we make of it? We need to secure a place for **A** along a gradient if we are to maintain that, for example, case **B** is more specific than **A** (assuming this is an intuitive reaction).

Thus, the problem is not simply that there may not be room for a sharp, categorical difference between switches and dials. If this were the extent of the issue, we could perhaps find reasons to settle for differences in degree. But my diagnosis is -the way I see it- more severe: even spelling out the difference is in degree becomes, conceptually speaking, a more elusive task than prima facie thought, as we now have degrees of two independent components of specificity which can pull in different directions.

At this point, one might think that a measure of causal specificity would solve (or dissolve) these worries. As a measure of causal specificity, SPEC is the most unmistakable construal of specificity as a quantitative property. But more importantly, it is expected to serve as a simple device for establishing the exact degree of specificity of any given cases, the (allegedly) least specific ones included. In fact, the measure can be applied to switch-like cases just the same**.** Far from any categorical difference between switches and dials, the measure yields a specificity value (a mutual information value, but where this is to be interpreted causally as specificity) in a common currency of bits, regardless of whether the cases under scrutiny were initially conceptualized as specific, nonspecific, more or less specific, dial-like, or switch-like. Yet, that the quantitative approach of SPEC solves (or dissolves) the issues proves to be a problematic claim, and the reason has to do, again, with the twofold nature of specificity.

In fact, if causal specificity is measured, as proposed by Griffiths and colleagues, by comparing the entropy of the probability distribution of the values of the effect variable *before* an intervention on the cause, and the entropy of the probability distribution of the effect variable *having performed the intervention* on the cause (this is, $\rm {H}\left(\rm {E}\right)-\rm {H}(\rm {E}|\widehat{\rm {C}})$), then, as shown by Griffiths et al. (2015) themselves, the following two cases **C** and **D** become indistinguishable (and both also indistinguishable from **A**):

They all yield a mutual information value of 1 bit, just like the switch case **A**. In case **C** we have an initial entropyBourrat of the effect of 1 bit, and because knowing the value of the cause leaves nil remaining uncertainty, the mutual information between $\widehat{C}$ and $E$ amounts to 1 bit. In the second case, **D**, the initial entropy of the effect is 2 bits (more values) and our uncertainty after knowing the value of the cause is not nil (either one of two values, thus 2 bits), so the mutual information value is exactly the same (1 bit). Yet, intuitively, we *may* want to say that they are different in important aspects^11^. The number of possible values for the causal variable is greater in **C** than in **A** and **D**; while the number of possible values for the effect variable is greater in **D**. In addition, in **A**, any change in the cause leads to a change in the effect, but not every change in the cause leads to a change in the effect in **C** or **D** (e.g., the change from $C={c}_{1}$ to $C={c}_{2}$).

In any case, the issue with the SPEC measure might be deeper than merely failing to account for the difference among presumably different cases. The quantity that would make the difference visible is the entropy of the source (Griffiths et al. 2015). However, this quantity plays no role in determining the specificity value. Recall the quantities relevant to SPEC from Sect. 3: the entropy of the probability distribution of the effect values, $\rm {H}\left(\rm {E}\right)$; the conditional entropy of $E$ having set the value of the cause by intervention, $\rm {H}(\rm {E}|\widehat{\rm {C}})$; and the mutual information between cause and effect having intervened on the cause, which obtains as $\rm {H}\left(\rm {E}\right)-\rm {H}(\rm {E}|\widehat{\rm {C}})$. The entropy of the source plays no role because our $\rm {C}$ here is always *an intervened *$\rm {C}$*,* this is, set to some value ${c}_{i}$ regardless of the possible repertoire we could have for the variable. All we need to measure specificity is the entropy of the probability distribution of the values of the effect variable *before* an intervention on the cause: $\rm {H}\left(\rm {E}\right)$, and the entropy of the probability distribution of the effect variable *having performed the intervention* on the cause: $\rm {H}(\rm {E}|\widehat{\rm {C}})$. Consequently, only $\widehat{\rm {C}}$ (but not $\rm {C}$) is relevant to the SPEC measure.

But the full range of possible values for either variable should matter, according to previous notions of specificity. Hence, SPEC misrepresents REPERTOIRE, in that the full range of possible values for $\rm {C}$ is at the end of the day irrelevant and all that matters is $\widehat{\rm {C}}$. For similar reasons, it also misrepresents CONNECTEDNESS, in that it only focuses on how one particular value of C maps with E value(s). This of course suggests that SPEC is not exactly a measure of causal specificity in any of the formulations in Sect. 4, but perhaps something different. It should be noted that others have discussed how to best implement the measure for particular comparisons (e.g. DNA and alternative splicing factors, Weber 2017b), but the measure itself has not been impugned or questioned as genuinely representing the philosophical idea of causal specificity.^12^ If my analysis is correct and the notion of specificity is indeed about CONNECTEDNESS and REPERTOIRE, then, I believe that SPEC is not an adequate approach as it distorts both components. In any case, assessing the adequacy of a measure for a given property requires, at the very least, that we have a good grasp of the property to begin with, and this is what I am most concerned with.^13^

Having identified and explained these issues with the very notion of causal specificity, we must take stock of the particular implications for causal parity. I discuss this next.

## Implications for the Causal Parity Dispute in Biology

Along the last few sections, I argued, contrary to what is assumed, that a quantitative view of specificity (either measurable or comparative) is not as intelligible and cannot be applied as systematically as it would be desirable for certain contexts –the causal parity quarrel in philosophy of biology being a salient case in point. I attributed this problem to the twofold nature of causal specificity, as REPERTOIRE and CONNECTEDNESS are independent conditions. Such independence makes it particularly problematic to deal with both components simultaneously and coherently.

What does this mean for specificity-based arguments and the CP thesis? I see two possible reactions. One is to think that causal specificity is not a single property but a mixing of two distinct ones that are, in fact, not even predicated of the same items. In this case, REPERTOIRE is a property of cause/effect variables, one about the range of variation that is possible for them. CONNECTEDNESS becomes a property of the mappings between causes and effects, corresponding to different types of functions (and perhaps non-functions as well). Each property by itself is perfectly coherent, so splitting causal specificity into two distinct (genuine) properties means a way around our issues. But splitting causal specificity into two independent properties means that we cannot compare causal relations from a single point of view. Parity and nonparity necessarily become relative to either independent property, something like “parity_REP_” and “parity_CON_”. This seems acceptable, but it does complicate things. What should we conclude from cases where we have two competing causal factors, each ranking higher than the other in one of these two properties? In addition, it could be argued that neither REPERTOIRE nor CONNECTEDNESS alone capture the intuition behind the comparison of the roles of DNA and the polymerase in protein synthesis. It seems that systematic application comes with the cost of giving up the original intuition.

Alternatively, we may want to retain the original intuition that certain causes, like DNA, are peculiar in that many different changes in the DNA lead to many changes in the effect, this is, some combination of both REPERTOIRE and CONNECTEDNESS. It seems that we do capture some interesting feature of genetic causation by such remarks, and there is no prima facie reason to disregard it. But, in turn, we may need to give up any pretension of systematic application, this is, that a concept of causal specificity (or a measure thereof) will lead to a systematic procedure whereby any two causal relations can be clearly compared and ranked in terms of their specificity if we wish to simultaneously take both components into account.

Let us consider the implications of the latter case: can we compare molecular causes from the point of view of specificity? This will sometimes be possible, for instance, if we compare two cases that differ along one component only but score the same along the other, but we will not always have clear, compelling intuitions regarding particular cases whenever this is not the case. Some comparisons might be more difficult than others, and will probably be influenced by the wider context and goals of the comparison. More important is another set of questions: Can specificity ground substantial philosophical claims about the ways they contribute to an effect? Can specificity settle the parity quarrel in one way or another? In which sense is it helpful to characterize DNA as a dial-like cause, and the polymerase as a switch-like one? In general, is it helpful to characterize biological causes as either dials or switches? I take to be an inevitable consequence of this analysis that we should rethink the meaning and scope of specificity-based philosophical claims. Showing that DNA or RNA molecules are more specific kinds of factors than polymerases or alternative splicing agents (hence supporting a claim of nonparity) is not as straightforward as it seems, and this is not (simply) due to biological facts. This need not mean that characterizing biological causes (or otherwise) as specific, nonspecific, or relatively specific is not helpful: causal specificity still picks out some feature of certain type causal relations, it just may not be the best argument in support or rejection of causal parity. What other causal concepts would do a better job in this respect is the topic of another paper.

## Conclusions

This paper undertook a deeper and encompassing exploration of causal specificity and the extent to which this notion can meet the goals that many have set for it. I first introduced the causal parity debate in philosophy of biology as the context of most discussions and elaborations on causal specificity, sketching the basic logic of specificity-based arguments against CP. Then, I reviewed several conceptualizations of specificity available in the literature. After raising the question as to what sort of property (categorical, quantitative) is specificity supposed to be, I laid out the rationale for a quantitative point of view. I argued, contrary to what seems to be assumed, that a quantitative view of specificity (either measurable or simply comparative), is not as intelligible and cannot be applied as systematically as it would be desirable. I attributed this problem to the twofold nature of causal specificity, as this property is about two logically independent conditions, REPERTOIRE and CONNECTEDNESS, which are not easily dealt with in tandem. The view in this paper is that, as long as such duality within the notion of causal specificity is not properly acknowledged, we cannot expect to arrive at anything but mixed and conflated claims that will not be particular profitable. I also contended that these are not minor issues, as they inevitably affect the very purposes for which specificity is invoked: *in general*, to compare/distinguish amongst causal factors; *in particular*, to settle the causal parity dispute in philosophy of biology.

## Footnotes

1 *Genetic factors* (or ‘resources’) are of course nucleic acid molecules, such as DNA/genes or RNAs. *Non-genetic factors* (or ‘resources’) is a broad label encompassing anything from various molecular factors (e.g. methylation patterns or diverse proteins), to cellular factors (e.g. maternal cytoplasmatic gradients or heritable structures, such as membranes), to heterogeneous environmental factors (e.g. exposure to language, incubation temperatures, or host-imprinting events). A case could be made that the ‘non-genetic’ label is a bit of an “anything goes”, but for the sake of the argument we need to take it at face value.

2 A couple of examples are provided, for instance, it is explained that “the facts of development” (read: the fact that there are distinct causal roles) do not justify assigning DNA the role of information and control while others get the role of material support for reading the information in DNA.

3 *Biochemical specificity* and *sequence specificity*, for instance, are two relevant notions for biologists. See (Griffiths and Stotz 2013, ch. 4; Stotz and Griffiths 2017) for an elaboration of the distinction.

4 Indeed, this working distinction between specificity as construed by biologists and specificity as construed by philosophers needs not assume that the two are unrelated (see for instance Lean 2020).

5 Woodward (2010) suggests a distinction between this idea (fine-grained influence, in his terminology) and another, quite vague idea of “one-cause-one-effect”. A causal relation one-cause-one-effect specific if only one *type* of cause produces only one *type* of effect, and it is not specific in this sense if different types of causes (e.g. smoking and exposure to asbestos) produce the same effect (e.g. lung cancer) or if the same type of cause (e.g. smoking) produces different effects (e.g. lung cancer and heart disease). I will restrict my analysis to the fine-grained influence sense of specificity.

6 In this framework, information seems to be given an epistemic interpretation, as reduction of uncertainty. The question arises as to whether this strategy de-ontologizes causal specificity.

7 This is called the “SPEC” measure by its proponents.

8 As to the range of variation that should be taken into account: all *possible* variation vs. some subrange, like *actual* variation; in *evolutionary time* vs. *synchronically.* As to the construal of the DNA causal variable: as the entire genome of *Drosophila*, vs. one particular DNA segment, such as the DSCAM gene. See (Griffiths et al. 2015 ; Weber 2017b) for details.

9 On the one hand, Griffiths et al. (2015) state that “Fine-grained influence requires both that the repertoire of effects is large and that the state of the cause contains a great deal of information about the state of the effect.” (p. 550). However, all that matters in this proposal is the entropy of the effect (not that of the cause). My own understanding of repertoire is not that the condition applies only to the effect but to both cause and effect. Thus, the distinction is not fully acknowledged in their work. (More on this in Sect. 6, where I discuss an issue with SPEC). On the other hand, Bourrat (2019) makes claims about two “dimensions” of specificity, but in a different sense. What he has in mind is Woodward’s distinction, in footnote 5, between one-cause-one-effect specificity and fine-grained influence. Because the information-theoretic measure captures only the latter, he argues, an additional measure is needed that would capture the former. My claim is different, as I am not concerned with one-cause-one-effect specificity. I contend that it is within the idea of fine-grained specificity (and slight variations thereof) that we can find significant ambiguity. However, he also claims that causal specificity amounts to *both* measures. This can be read in two very different ways. If it means that both INF and one-cause-one-effect are equally legitimately causal concepts, to which he also subscribes explicitly, I see no problems (although I do find it misleading to speak of “amounting to”). If, on the other hand, he means that there really is a single notion of causal specificity that is really a combination of two ideas, then having separate measures for each would become a problem unless there is also a way of articulating the two (especially on the conceptual level). For this reason, I am inclined to the former interpretation.

10 When the type of connection is bijective, CONNECTEDNESS does say something about REPERTORIRE, in some sense. It tells us that the sets will be the same size, but that is the extent of it. It remains true that a bijection will not imply that this size is 5, 138, or 38,016.

11 In case **C**, we are ignoring the reasons for splitting the cause in such a way that (some) values are individuated which do not make a difference for some values of the effect. This would require, in part, considering how a particular theory of causation deals with granularity or level of description. Case **D** can be thought of a case of causal indeterminacy. Unlike Griffiths et al., Woodward does not consider this case, as **D** does not represent a function. Perhaps it is assumed that the relevant causes in biology are deterministic. How common or rare are instantiations of these structures is a different question.

12 Without impugning SPEC per se, Bourrat (2019) claims that it is of a limited application as it fails to measure one-to-one specificity. While I agree with Bourrat that SPEC is at best partial and fails to capture all there is to be quantified over, I disagree that it provides a good representation of *at least one* of the two senses of causal specificity (Woodward’s INF).

13 A reviewer suggested to consider whether SPEC’s emphasis on the probability distribution suggests a third component of specificity. This is an interesting suggestion, but I have some doubts about this way of looking at the role of probabilities here. I believe that while probabilities are relevant in general, they are not better viewed as a further component of specificity. I take that the reviewer refers not to the conditional probability of obtaining an effect given a cause, but to individual probabilities of the values of the cause/effect variables. For instance, the probability of me setting the tuning dial on position *p*_*i*_, the probability of me setting the dial on position *p*_*j*_, and so on. Now, I think that the characteristics of the causal structure as a whole are independent of individual probabilities in the sense above, at least, if we take available definitions of specificity. The way to think about the different states here should be rather counterfactual: if I were to set the dial on position *p*_*i*_, then, radio station *r*_*i*_ would obtain. It does not matter how unlikely it is for me to do that for characterizing the causal structure. It seems to me that the probability of occurrence of *p*_*i*_ which, suppose, is very low, would not by itself affect an ascription of specificity to the extent that one concedes the truth of the conditional ‘if I were to set the dial on position *p*_*i*_, then, I would tune radio station *r*_*i*_’. In my view, individual probabilities are irrelevant to analyzing the causal specificity of a given causal structure, because specificity is not about any particular state but about the overall structure. However, this is not to deny that probabilities are relevant to the SPEC measure. Indeed, they are absolutely relevant to SPEC, insofar as they are essential to information theory and SPEC uses such formalism. But I believe that we should not draw the wrong conclusions from this fact for the simple reason that the measure might be failing to capture the notion of specificity (as I have suggested). Thus, I believe that that the key, distinct components of specificity are the two I have put forward.
